# On Peri-Implant Bone Loss Theories: Trying To Piece Together the Jigsaw

**DOI:** 10.7759/cureus.33237

**Published:** 2023-01-01

**Authors:** Eduardo Anitua, Mohammad H Alkhraisat, Asier Eguia

**Affiliations:** 1 Regenerative Medicine Laboratory, Instituto Eduardo Anitua, Vitoria, ESP; 2 Estomatology II, University of The Basque Country (Universidad del País Vasco, UPV/Euskal Herriko Unibertsitatea, EHU), Leioa, ESP

**Keywords:** peri-implant diseases, foreign body, dental implants, peri-implantitis, marginal bone loss

## Abstract

This review aims to explore the plausibility of new theories on the etiopathogenesis of marginal bone loss (MBL) and peri-implantitis (PI) and to discuss possible underlying pathogenic mechanisms. The former concept of osteointegration of dental implants can now be conceptualized as a foreign body response histologically characterized by a bony demarcation in combination with chronic inflammation. Different risk factors can provoke additional inflammation and, therefore, pro-inflammatory cytokine release in soft tissues and bone, leading to an overpass of the threshold of peri-implant bone defensive and regenerative capacity. Progressive bone loss observed in MBL and PI is ultimately due to a localized imbalance in the receptor activator of nuclear factor kappaB ligand (RANKL)/Receptor activator of nuclear factor κ B (RANK)/osteoprotegerin (OPG) pathway in favor of increased catabolic activity. The genetic background and the severity and duration of the risk factors could explain differences between individuals in the threshold needed to reach an imbalanced scenario. MBL and PI pathogenesis could be better explained by the “inflammation-immunological balance” theory rather than a solely “infectious disease” conception. The link between the effect of biofilm and other risk factors leading to an imbalanced foreign body response lies in osteoclast differentiation and activation pathways (over)stimulation.

## Introduction and background

Osseointegration term was first used by Brånemark in 1976 and then defined as direct contact between implants and bone at the resolution level of the light microscope [1]. The notion of a simple wound healing encompassed with a sort of chemical attachment was linked to the assumption of titanium being bioinert, without any adverse tissue reaction [2]. Donath et al. foresighted for the first time an alternative explanation and claimed that osseointegration was but a foreign body reaction embedding the implant in the bone as a mode of protection for nearby tissues [3].

Recent publications have brought about a paradigm shift in the definition of both osseointegration (now conceived as a chronic low-grade or “balanced” foreign body response) and peri-implant pathology (now conceived as a primary immune imbalance rather than a purely infectious disease) [2,4-8]. Dental implants' survival depends on an equilibrium between the defense reactions in the forms of chronic inflammation and activation of the innate immune system [4]. Osseointegration has been recently re-defined as “a foreign body reaction where interfacial bone is formed as a defense reaction to shield off the implant from the tissue” [2].

Marginal bone loss (MBL) and peri-implantitis (PI) are two clinical conditions characterized by progressive bone loss, the former sometimes (but not always) preceding the latter. This localized bone loss as in the case of periprosthetic osteolysis or “aseptic loosening” in other bone sites outside the mouth, is caused by a local increase in resorptive activity and/or reduced new bone formation [9]. Possibly, the effects of different known risk factors for MBL and PI contribute to the trespassing of an individual genetically influenced threshold, which locally disrupts the physiological bone turnover. Oral biofilm could act as the main partner (not strictly necessary) to overpass the threshold and initiate the imbalance of the so-called “foreign body equilibrium” or may interact secondly leading to infection and further worsening the problem in progress.

## Review

Immunological imbalance: impaired bone turnover

Bone health relies on a delicate balance between old bone resorption and new bone formation namely bone turnover, which is driven by a constant interaction between cells from the immune and skeletal systems. Ultimately this phenomenon is controlled by the crosstalk and coordination between osteoblast and osteoclast lineage cells [9]. Communication between bone-forming and bone-resorbing cells ensures an integrated response of bone tissue to systemic, local, or mechanical stimuli [10]. Normal bone remodeling or turnover occurs in small regions of bone asynchronously throughout the skeleton: bone remodeling unit (BRU)/basic multicellular unit (BMU). This process is characterized by five well-defined phases: (1) activation of remodeling, (2) bone resorption, (3) reversal phase, (4) bone formation, and (5) quiescence [9]. During the activation phase, the osteoclasts are recruited. The bone resorption starts during the resorption phase and subsequently. In the reversal phase, the osteoclasts undergo apoptosis, and the osteoblasts are recruited. At this point, the formation phase begins, and the osteoblasts lay down a new organic bone matrix that is lately mineralized. This cycle concludes with a new quiescence period. Therefore, bone remodeling is a process that requires crosstalk and sequentially works in the same BRU of osteoclasts and osteoblasts [10,11].

When the amount of new bone formed by osteoblasts is not matched to the amount of old bone resorbed by osteoclasts, bone mass changes. A greater resorption than formation leads to bone loss [9]. At the systemic level, this circumstance is common during aging, but when this negative balance is excessive, it is also the origin of different pathologies (e.g., osteomalacia, osteoporosis, and diverse bone diseases). On the contrary, defective bone resorption also turns in pathologic conditions (e.g., osteopetrosis) [12].

At the local level, mechanical overload, inflammation, neoplasms, foreign body reactions, and others could lead to focal changes in this physiological turnover leading to bone resorption [13-15]. The periodontal and peri-implant bone is not an exception and different well-known risk factors for MBL and PI can cause local inflammation ultimately leading to osteoclast precursors chemotaxis, differentiation, and (over)activation [16,17]. The synergistic effect of different risk factors for MBL and PI [18], systemic conditions [19], and hereditary predisposing factors [20] could provoke or aggravate an imbalanced bone turnover and may promote secondary infection that feedbacks the inflammatory response.

The conversion from a state of inflammation contained in the soft tissue (e.g., peri-implant mucositis) to a state of inflammation involving the loss of peri-implant marginal bone (e.g., PI) has been assumed to occur, but the mechanisms underlying this shift are not fully understood [14]. Probably, the synergistic effect of different factors (related to the implant, the patient, and the prostheses) is needed to trespass a person-specific threshold influenced by systemic conditions or diseases and genetic background. This could be the basis to explain the different clinical responses observed between patients when facing similar microbiological insults.

The actual role of risk factors for MBL and PI

Although it was widely claimed that the most common reason for MBL is infection [14,21,22], MBL is not primarily related to biofilm-mediated infectious processes as in the pathogenesis of periodontitis [5,23]. Initial marginal bone resorption could represent a reaction to treatment instead a disease process [5,23,24], but it could be aggravated by the same factors that contribute to PI development. Once severe MBL has developed, a secondary biofilm-mediated infection may follow [23]. The progression from MBL to PI does not necessarily always take place and a disrupted “foreign body equilibrium” can be re-established [5,23]. Even when a PI occurs, successful treatment can lead to recovery of the “foreign body equilibrium” [5,23]. In such a situation, peri-implant health status can be recovered, occasionally with complete bone regeneration. Nevertheless, when PI progression ceases, in most cases the lost bone is not fully regenerated. This clinical situation has been recently defined as “peri-implant health with reduced implant support” [25].

Biofilm formation, maturation, and proliferation of perio-pathogenic species on prosthetic and implant surfaces are well-evidenced risk factors for peri-implant disease initiation and or worsening [18,26]. However, all peri-implant diseases cannot be solely explained as a result of a microbiological insult [14]. Probably, in addition to microbiological challenges, numerous local and systemic factors contribute to the onset and progression of peri-implant diseases [18,27,28]. Therefore, the therapeutical approach to these conditions shouldn´t be solely based on an attempt to eliminate/reduce the so-called “bacterial load”. Instead, the prevention and management of peri-implant diseases should be ideally more prone to deal also with other involved factors such as the prosthetic design and fitting, implant surface, design and positioning, distribution of occlusal loads, or patients´ systemic conditions, among many others.

Beyond a struggle against the plaque, a paradigm shift should be considered to properly focus the treatment goal on preventing and/or reducing the existence of a proinflammatory microenvironment surrounding the implant. To eliminate/reduce the presence/amount of pathogenic bacteria, using surgical and non-surgical approaches is certainly beneficial [29]. However, the crux of the matter is to avoid osteoclast (over)differentiation and (over)activation, which in fact is provoked by the local release of proinflammatory cytokines, different cell proliferation factors, and other molecules [30-33]. The overexpression of these proinflammatory mediators results not only from the perio-pathogenic bacterial colonization and activity [33,34], but also as a result of other risk factors such as occlusal overload, presence of cement remnants, poor prosthetic fitting and micromovement, corrosion, and many other factors that are later discussed [13,35,36] It should be also noted at this point, that the insult caused by the oral biofilm is due to complex interactions between bacteria, viruses, fungi, archaea, and protozoa [31,37] and not exclusively by perio-pathogenic bacteria. This should be considered when selecting a supportive antimicrobial treatment. The most reported bacteria associated with PI are obligate anaerobe Gram-negative bacteria, although Gram-positive rods and other Gram-positive species could be also involved [27,37,38]. The immune response is triggered by the dysbiosis of the oral microbiota [37]. Peri-implant microbiota and its metabolites can induce the production of proinflammatory cytokines such as IL-1, IL-6, IL-17, tumor necrosis factor alpha (TNF-α), macrophage inflammatory protein (MIP-1), and macrophage chemoattractant protein (MCP-1) by different immune cells, including neutrophils, monocytes, macrophages, dendritic cells, T cells, and B cells, leading to an increased expression of receptor activator of nuclear factor κ B ligand (RANKL) [37-43]. Lately, this enhances locally the osteoclast differentiation and function through the RANKL/receptor activator of nuclear factor κ B (RANK)/osteoprotegerin (OPG) pathway [44], thus increasing the bone resorptive activity [45]. It is noteworthy that higher levels of proinflammatory cytokines (IL-1, IL-6, IL-8, and TNF-α) in the crevicular fluid (CF) of implants with PI have been observed by different authors than in healthy implants [46-49]. Irshad et al. observed that fibroblasts from PI and periodontitis lesions expressed higher levels of interleukin (IL)-1β, IL-8, and MCP-1 than fibroblasts from periodontally healthy individuals [50]. Duarte et al. found that IL-12 and TNF-α mRNA levels were higher in severe PI than in Initial PI and in peri-implant mucositis [51]. Differences in the soluble RANKL (sRANKL) concentration and RANKL\OPG ratio in the CF from PI and healthy sites have been also noticed [17,52]. Inflammatory mediators, such as IL-1β or TNF-α have been proposed to be useful as additional criteria for a more robust diagnosis of PI [48], but unfortunately, differences in inflammatory cytokines or bone regulation markers (in CF concentration or tissue expression) are not specific enough (until what is yet known) to be individually used as risk biomarkers for PI or to evaluate the disease evolution [53].

**Figure 1:**
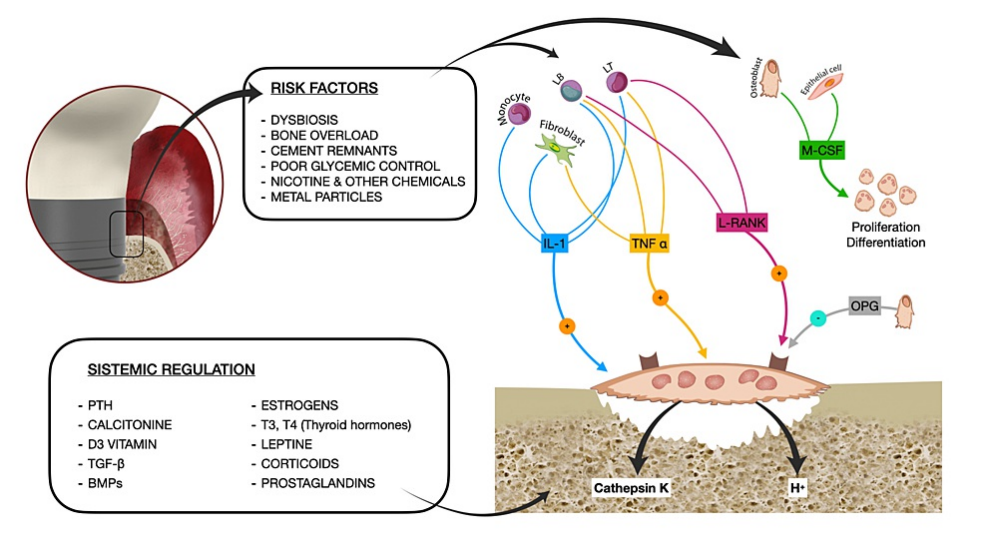
Factors that may influence osteoblast activation Different risk factors for PI and MBL may lead to the overexpression of macrophage colony-stimulating factor (M-CSF), Receptor activator of nuclear factor κβ ligand (RANKL), tumor necrosis factor-α (TNF- α), and Interleukine-1 (IL-1). These cytokines along with other systemic mediators govern osteoclastic differentiation, proliferation, and activity. MBL: marginal bone loss; PI: peri-implantitis Image credit: Eduardo Anitua

Figure 1 depicts the summary of factors that may influence osteoblastic activity at the peri-implant level, and thus the bone apposition-reabsorption balance.

The 2017 World Workshop on the Classification of Periodontal and Peri-Implant Diseases and Conditions [18] emphasized the infectious nature of PI [14]. It was concluded that there is strong evidence of an increased risk of PI in patients with a history of chronic periodontitis, poor plaque control skills, and no regular maintenance care. Despite this, a possible misinterpretation of some “ligature experiments” [14] has probably led many clinicians to focus on “infection-centered theories” and to underestimate the additional role of other factors in the etiology of peri-implant diseases.

Genetic factors

There is an increasing number of observational studies that have addressed a potential association between various gene polymorphisms (most of them single nucleotide polymorphism (SNPs)) in genes modulating inflammatory responses (IL-1, IL-4, IL-6, IL-17, TNF-α) or bone metabolism (RANKL, OPG) and peri-implant biological complications [20,54]. Although the results vary according to the ethnicity of the studied population and the small sample size of the studies [20], the available evidence points to a potential influence of some SNPs in the pathogenesis of PI [18].

Some functionally relevant polymorphisms in the genes that encode pro-inflammatory cytokines can condition an overproduction and even a basal increase in the production of the respective cytokines [55,56]. This fact could modulate the local activation of the immune cells when they detect pathogen-associated molecular patterns (PAMPs) and damage-associated molecular patterns (DAMPs) [57]. This phenomenon has been linked to a wide range of systemic disorders, in which there is an altered inflammatory response, such as rheumatoid arthritis, psoriasis, or inflammatory bowel disease [55,56,58].

At the oral level, different polymorphisms in the IL-1 gene cluster have been widely studied in relation to periodontitis [59] and augmented risk for early dental implant failure [60] or PI [61]. Gruica et al. also observed a statistically significant synergistic effect between the presence of a risk allele in the IL-1 gene cluster and heavy smoking with an increased risk of PI [62]. TNFα is another key proinflammatory cytokine in the early stages of the inflammatory response [63]. Rakic et al. observed that the presence of an AG genotype in the polymorphic region of TNFα (308-) was related to a five-time higher risk for PI, while a GG genotype was more prevalent in healthy individuals and conferred a certain “protective effect” [64].

RANKL is a key molecule in the regulation bone turnover through the RANKL/RANK/OPG pathway, produced mainly by osteoblasts and fibroblasts [10-12]. Upon binding of RANKL to RANK (its transmembrane receptor) activated osteoclasts acidify their environment (destabilizing the inorganic bone component) and release matrix metalloproteinases (MMPs) and Cathepsin K (which disrupts bone organic components). Different SNPs in RANKL [65,66], OPG [67], and MMP-1 [68] have been related to PI and implant failure.

The real role of genetic polymorphisms in the pathogenesis of PI and other peri-implant conditions will probably be conclusive in the future if new studies are performed in different ethnic-origin populations and with larger sample sizes. Also, analyzing possible synergisms in patients carrying different of the above-mentioned SNPs could be of interest in this way. There is strong evidence that a history of periodontitis constitutes a risk factor for PI in the same patient [18]. Since many identical genetic conditions have been described as risk factors both for PI and periodontitis [69-72], it is debatable to what extent this relationship is a result of microbiological factors or genetic factors (or both).

Smoking habit

There is currently no conclusive evidence that smoking constitutes a risk factor for PI [18] but this does not mean that smoking has no negative influence on bone loss around dental implants. To properly analyze this factor could be challenging, since confounding factors such as periodontitis or concurrent alcohol intake may hinder the interpretation of the results. The remaining effects of smoking on ex-smokers and accurate quantification of smoking habit can add further complexity as well. Cigarette smoking has a negative impact on bone; it is considered a risk factor for the development of osteoporosis, disturbs the bone healing process, and prolongs the healing time after fractures [73]. At the oral level, chronic smoking habits modulate peri-implant cytokine profile in clinically healthy implants [74]. Although the oral negative effects of smoking are not fully understood, It has been observed that smokers had an increased risk of complications, including infection, implant loss, mucositis, and PI, compared with nonsmoking patients [75]. Mustapha et al. observed that Implants placed in smokers presented a 140.2% higher risk of failure than implants placed in non-smokers [76]. In a recent meta-analysis by Hadadi et al., implant success rate was higher in nonsmokers than smokers (OR= 0.43, 95%CI = 0.26-0.72, P < 0.0001) and smoking habit seemed to affect the implant survival rate and MBL negatively [77].

Smoking can reduce bone formation rate and decrease bone mineral density [78], increase the release of free radicals [79] and impair angiogenesis (in early bone healing) [73,80]. Moreover, nicotine increases the production of inflammatory cytokines (IL-6, TNF-α) by osteoblasts [81] and cigarette smokers present higher levels of IL-1 and MMP-9 than non-smokers in peri-implant gingival crevicular fluid [82]. Therefore, nicotine and chemicals in tobacco smoke could contribute to enhancing a pro-inflammatory micro-environment in both soft and hard tissues surrounding dental implants and act in synergy with other risk factors to alter the “foreign body equilibrium”.

Diabetes and other systemic conditions

The evidence as to whether diabetes is a risk factor for PI was considered “inconclusive” in the 2017 World Workshop on the Classification of Periodontal and Peri-Implant Diseases and Conditions [18]. Nevertheless, studies reporting results regardless of the level of glycemic control should be carefully interpreted [83]. Poor glycemic status (PGS) may negatively modulate bone factors (OPG, transforming growth factor beta (TGF-β), osteopontin, osteocalcin) during healing and seems to be an important factor affecting implant complication rates, including MBL [84]. Probably, PGS and not the fact of being diabetic is what jeopardizes dental implant treatment success. In animal models, glycemic fluctuation may aggravate inflammation and bone loss in PI caused by dysbiosis and the activation of TLR2/4-IRAK1-TRAF6 signaling of the innate immune system [85]. In humans, changes in peri-implant microflora have been observed in patients with type-2 diabetes [86], particularly in deep pockets [87]. Peri-implant inflammatory variables (plaque index, bleeding on probing, probing depth, and MBL have been shown to be worse among cigarette smokers and non-smokers with type-2 diabetes than non-smokers without diabetes, hyperglycemia being a stronger mediator of inflammation than cigarette smoking [88].

Turning back to what was stated before, the RANKL/RANK/OPG pathway is the "bottleneck" regulator of osteoclastogenesis and bone resorption, both in physiological and pathological conditions [89]. Concentrations of RANK, sRANKL, OPG, and sclerostin are significantly increased in the crevicular fluid of patients with PI compared with healthy patients [90]. RANKL/OPG ratio could be further up-regulated in smokers and diabetics [89]. Thoroughly understanding of the complex relationship between PI, MBL and diabetes may require controlling in new studies the action of antidiabetic medications and their possible role as confounding factors. For example, it has been observed in vitro that metformin could inhibit osteoclast formation and activation (downregulating RANKL, M-CSF genes, and osteoclast fusion gene DC-STAMP) [91].

Prosthetic factors

The influence in the pathogenesis of PI and MBL of prosthetic design and materials has probably been underestimated in the past, therefore corrections or modifications to prostheses were not always included in prevention and/or treatment protocols for MBL and PI. Beyond iatrogenic factors such as inadequate seating of the prosthesis [92], there is increasing evidence that the surface features (both biocompatibility and resistance to bacterial adhesion) of the abutment [93,94], the type of prosthesis [95], or even the design of the emergence profile (prosthetic contour) [95-98] may also play an important role.

The quality of implant-abutment connection (IAC) is well-known to be a key factor that influences MBL and the risk for PI, as micro-gaps represent a site for dental plaque aggregation and maturation [99,100]. Micro-gaps and even the inner part of the implant, once colonized, may constitute a reservoir that could subsequently contaminate implant surroundings and interfere with peri-implant tissues' health [99,100]. Micro-leakage through the micro-gaps can favor a pro-inflammatory micro-environment at the level of the IAC as it can carry to the tissues not only bacteria but also their metabolism products, exotoxins, wear debris (metal particles) and remnants of the bacterial walls (such as lipopolysaccharide (LPS)); i.e. a combination of immunogenic substances [99-102]. Also, micromovement (linked with a lack of perfect fitting) [101] and abutment disconnection and reconnection [103] can further aggravate this problem contributing once again to disturbing the “foreign body equilibrium”.

**Figure 2:**
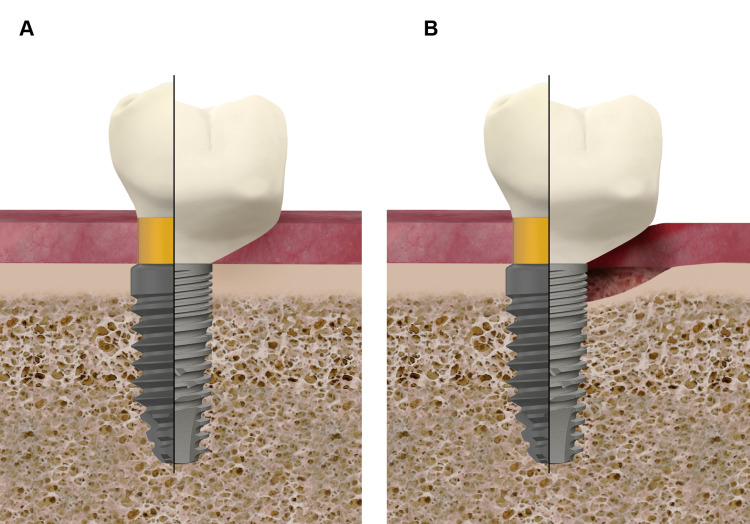
Influence of the emergence profile on the risk of MBL/PI (A) Implant loading time. Please note the reduced connective tissue thickness when the emergence profile is far >30° and/or convex (like in the clinical situation in the right part of the figure). (B) Over time, the risk of MBL and PI has been stated to be higher in the clinical situation on the right-hand side of the figure. MBL: marginal bone loss; PI: peri-implantitis Image credit: Eduardo Anitua

Until recently, the importance of the design and the shape of the subgingival part of abutments and prostheses was poorly understood. In this sense, Katafuchi et al. observed that emergence angles (between the prosthetic contour of the prosthesis and the axis of the implant) of >30° were a significant risk indicator for PI and that convex profiles created additional risk for bone-level implants [96]. This was also observed in a cross-sectional study carried out by Yi et al. [97]. Majzoub et al. found that marginal bone loss in the early post-diagnosis period (one year) of PI, could be affected by the restoration emergence angle and shape too [98]. It has been claimed that higher restoration angles and convexity may hinder proper hygiene measures due to limited access [96-98]. This rationale has been assumed without clear evidence, but what seems to be more reasonable to explain this relation between the shape and MBL or PI, is the fact that the higher the emergence angle (measured from the prosthetic platform in bone-level implants and from the supracrestal part of tissue-level implants) the less the thickness of connective tissue over the cortical bone in direct contact with the implant, thus hindering its blood supply (Figure 2).

The same might be applied to the relation between abutment height and MBL observed by many authors [104]. Taking into account that the design of the abutments in most of these studies was “straight” (emergence angle 0° along all the abutment height) [105-107], the higher the abutment, the higher the thickness of the connective tissue, the better the blood supply for the cortical bone in contact with the implant neck. This is interestingly consistent with the clinical observation that the higher abutment, the less MBL and PI risk, with a critical cut-off point over 2mm [104-107].

The role of occlusal loading as a risk factor/indicator for MBL or PI has not been yet definitively evidenced in the literature [18] and some controversies remain. But once again, the lack of a high degree of evidence studies is not to say that occlusal loads do not influence MBL and/or PI onset and progression. Within a still undefined “ideal threshold” (probably variable in each individual), occlusal forces transmitted to the bone may enhance both osteointegration and the features of the bone in close contact with the implant [108,109]. Probably, in absence of a clear definition of what exactly is “excessive occlusal load” [109] and given the complexity of exactly measuring in vivo the real strength and direction of occlusal loads in every single implant, it is difficult to clarify this point by now. Nevertheless, the loads transmitted through the implant-bone contact have lastly an influence on the RANKL/RANK/OPG pathway through the so-called “mechano-transduction phenomena” [108]. Interestingly, in a recent prospective study, the authors observed changes in the expression of cytokines from peri-implant CF (TNF-α, IL-10, IL-6, IL-1β, IL-8) in the first year after an occlusal adjustment [110].

Bio-Tribo-corrosion and metal particles and ions released from implants or implant-prosthesis interfaces could contribute to MBL and PI pathogenesis and seems to negatively influence implant survival [111-113]. The release of wear debris can trigger or aggravate inflammation in peri-implant tissues through foreign body reactions or local dysbiosis [111-113]. In addition to provoking the release of pro-inflammatory mediators, metal ions could have direct effects on osteoblastic cell viability, apoptosis, and regulation of bone-resorbing mediators [114]. Similar pathogenic effects on PI and MBL could be attributed to cement excesses and the release of cement particles [115-116].

Surgical factors and implant positioning

Wrong positioning of implants can make it difficult to perform oral hygiene and maintenance, and therefore cause dysbiosis which fosters inflammation in the surrounding tissues [18,112]. Also, implant size may condition the amount of bone around the implant and its vascularization [117]. Furthermore, among other factors, wrong implant positioning can result in a lack of keratinized mucosa; a condition that may influence the clinical behavior of the implants [18,118]. All these circumstances may hinder the maintenance of the “foreign body equilibrium” too.

There are a number of other factors that have been related to MBL and PI, such as excessive bone compression during placing [119], micromotion of the implant [119,120], and many others. Unfortunately, all of them cannot be included in the extent of this critical review. When analyzed in detail, most of them finally lead to the same “bottle of neck”; the RANKL/RANK/OPG pathway. Probably, MBL and PI are the cause of the interplay or synergy between patient-related, implant-related, biofilm-related, prosthesis-related, and surgical-related factors. An individual genetic background may modulate the response to these factors and bias the threshold between health and disease.

## Conclusions

MBL and PI are conditions with multifactorial and complex pathogenesis. The “inflammation-immunological balance” (or foreign body equilibrium) theory could help in better clarifying how these conditions occur rather than “purely infectious disease” theories. Furthermore, a less restrictive conception may enhance the preventive and therapeutic protocols, also focusing on missing factors due to the lack of high strength of evidence by now, but with plausible importance. Ultimately, focusing on preventing osteoclast resorptive activity may be more reasonable and advantageous than focusing solely on decontaminating surfaces. The link between risk factors leading to a dis-balanced foreign body response lies in osteoclast differentiation and activation pathways (over)stimulation.
